# Values and attitude certainty: The case for attitude clarity and correctness

**DOI:** 10.3389/fpsyg.2022.975864

**Published:** 2022-11-10

**Authors:** Kevin L. Blankenship, Kelly A. Kane, Marielle G. Machacek

**Affiliations:** Department of Psychology, Iowa State University, Ames, IA, United States

**Keywords:** attitude certainty, attitude clarity and correctness, value relevance, resistance to persuasion, intentions

## Abstract

Three studies examined how the perception that one’s attitudes are based in values affects attitude clarity and correctness. Specifically, perceiving that one’s attitude is based in important values increases attitude clarity (the subjective sense that one knows one’s attitude) but not attitude correctness (the subjective sense that the attitude is correct). To test this, participants read a counterattitudinal message and were given feedback about the basis of their attitude. Relative to participants who learned that their attitudes were weakly based in values, participants who were told that their attitudes were strongly based in values reported greater attitude clarity than correctness (Study 1). Similarly, increases in attitude clarity from having an attitude based in values increased the perception that participants effortfully processed the message (Studies 2 and 3), the belief that participants more successfully resisted the message, and participants’ intentions to act on the attitude.

## Introduction

“For me, human rights simply endorse a view of life and a set of moral values that are perfectly clear to an 8-year-old child. A child knows what is fair and isn’t fair, and justice derives from that knowledge.”

—*Tom Stoppard*

Imagine that your opinion toward an important issue is met with challenge by an opponent. Assuming that you wanted to continue to hold your current opinion, how might you take on this challenge and minimize its impact? Now imagine that your opinion is based in a set of values that are expressed through holding this opinion. How might these underpinning values affect your ability to take on this challenge?

Such a situation has been a topic of interest in the social sciences for decades (Vaughn and Mangan, 1963; Rokeach, 1973). An evaluation’s association with important values has long been viewed an important consideration in the determining the strength of that evaluation (Rokeach, 1968). After all, values are “desirable, trans-situational goals, varying in importance, that serve as guiding principles in people’s lives” (Schwartz, 1992, p. 122; see also Rokeach, 1968; Maio, 2010). It is no surprise then that attitudes based in or expressed by important values are attitudes with enhanced properties (e.g., personal importance; Boninger et al., 1995; certainty; Gross et al., 1995) that persist, resist change, and guide behavior. The current research examines an attitude’s association with important values as an attitude strength feature. In particular, we focused on a value’s perceived link to an attitude and its ability to influence attitude certainty, a key dimension of attitude strength.

### Values and attitude certainty

Values can impart strength to associated attitudes (Sherif and Sherif, 1967; Rokeach, 1968; Homer and Kahle, 1988; Holbrook et al., 2005) and relevant behaviors (e.g., Hertel and Kerr, 2001; Maio et al., 2009). However, relatively little is known about the psychological mechanisms that explain an attitude’s increased strength from its association with values. From a structural perspective, one could consider attitudes toward an issue and its supporting values as part of an attitude system (i.e., interattitudinal structure, Abelson and Rosenberg, 1958; McGuire, 1989). Values come with extensive cognitive structures connecting them to a variety of beliefs and specific attitudes (Schwartz, 1992). Therefore, attaching an attitude to a value can create a relatively strong attitude, especially through overcoming direct attacks on that attitude (Scott, 1969; Eagly and Chaiken, 1995, 1998). Thus, the structural support that an attitude receives from a value can impart strength on that attitude (Bernard et al., 2003). Consistent with this perspective, increasing the structural strength of a value (i.e., accessibility) changes other attitudes linked to the value (Blankenship et al., 2015). Changing an individual’s values can also change their closely linked attitudes.

Alternatively, the perception that one’s attitudes are based in important values may buttress perceptions of the attitude’s strength, such as attitude certainty, the subjective sense that one’s attitude is valid (Gross et al., 1995; Rucker et al., 2014). Increased confidence has been associated with increased use of the cognitive contents that are held with confidence (Petty et al., 2007). Moreover, undermining one’s confidence in a value has been shown to undermine support for an attitude related to that value (Blankenship et al., 2012). Thus, if a person perceives that values provide a basis for their attitudes, then they may hold those attitudes with greater confidence. Indeed, attitudes linked to values tend to be held with greater certainty (Johnson and Eagly, 1989; Maio et al., 2009).

Increases in attitude certainty also have important consequences for other attitude-relevant appraisals associated with attitude strength. That is, in addition to increasing information processing (Wan and Rucker, 2013) and persuasion resistance (Tormala and Petty, 2002), attitude certainty can also affect the appraisals individuals make independent of actual processing and resistance (see Rucker et al., 2014, for a review). For example, relative to low-certainty attitudes, attitudes held with greater certainty are associated with the perception of having thoughtfully considered attitude-relevant information (Barden and Petty, 2008; Wan et al., 2010), of being based on high-quality supporting thoughts, and of resistance to change; despite attitudes not actually changing in favorability (Tormala et al., 2006). Thus, attitude certainty may be part of a larger sequence of metacognitive appraisals that have implications for attitude strength.

### Clarity or correctness?

Although prior work has established the connection between value-attitude relations and certainty, less research has focused on what certainty means when values support or express one’s attitudes. The current research seeks insight into the relation between value support and attitude certainty. Recent work guided by a multifactor model of attitude certainty has examined two subcomponents of attitude certainty (Tormala and Rucker, 2007). Attitude clarity is the subjective sense that one truly knows one’s own attitude, whereas attitude correctness is the sense that one’s attitude is valid (Petrocelli et al., 2007). While related, the two facets have independent influences on global measures of certainty, as well as different antecedents and outcomes. For example, Petrocelli et al. (2007) found that repeated expression of an attitude affects perceptions of that attitude’s clarity but not its correctness, in part because expressing an attitude increases perceptions that one knows what one’s attitude is. Similarly, being listened to when sharing an opinion increases speakers’ attitude clarity but not correctness (Itzchakov et al., 2018). Alternatively, emphasizing social support of an attitude (e.g., social consensus) affects perceptions of correctness more than clarity because consensus justifies an attitude (Visser and Mirabile, 2004). Attitude correctness also predicts a competitive conflict style (Rios et al., 2014) and more negative attitudes toward outgroup members when group status is salient (Roth and Rios, 2022), compared to attitude clarity. Thus, while clarity is enhanced by opportunities to “know,” express, or rehearse one’s attitude, correctness is enhanced by external validation, such as consensus, social comparison, and ingroup-enhancing outcomes.

The current set of studies examines which aspect of certainty may be primarily tied to a value’s influence on attitudes. Is it the case that the perception one’s attitudes are based in values increases the perception one knows one’s attitude (i.e., attitude clarity), or might it be that value support is driven by the perception that one’s attitude is valid (i.e., attitude correctness)? The answer may lie in how values and their related motivations have been conceptualized over the decades. While values are culture-specific socially construed guides for behavior, they do become embedded in and linked to the self over time (Rokeach, 1968; Kochanska and Thompson, 1997; Mischel and Morf, 2003). Many definitions of value-related phenomena in psychology emphasize a value’s link to the self (Sherif and Cantril, 1947; Rokeach, 1968; Zunick et al., 2017); one’s values are a central aspect of the self (Petty and Cacioppo, 1990; Hitlin, 2003) and serve to affirm the self when it is threatened (Steele, 1988). This internalization may then increase clarity of the self and self-relevant phenomena such as attitudes. Attitude clarity is more closely linked to one’s subjective, personal expression of attitudes; therefore, it would make sense that attitudes closely connected to values become validated through increased clarity, rather than increased correctness.

Research and theory in attitudes and persuasion has examined the link between attitudes and the self, with values conceptualized as a conduit through which attitudes are formed and maintained (Vaughan-Johnston et al., 2022). For example, Social Judgment Theory (Sherif and Sherif, 1967) conceptualizes value-relevant attitudes as a part of the self-concept; such attitudes are created by the “activation of attitudes linked to important values” (Johnson and Eagly, 1990 p. 290, 1990). Similarly, functional theories of attitudes (Smith et al., 1956; Katz, 1960) posit that value-expressive attitudes help establish self-identity (e.g., “central” attitudes; Rokeach, 1968; Pomerantz et al., 1995; Clarkson et al., 2009; see also Abelson and Prentice, 1989). In fact, value-expressive attitudes are those that provide “clarity to the self-image” (Katz, 1960, p. 1975).

We therefore posit that, in general, an attitude’s link to values is more likely to strengthen that attitude by increasing attitude clarity than attitude correctness. Whereas external forces such as consensus and social comparison seem responsible for judgments of attitude correctness (Petrocelli et al., 2007), having an evaluation linked to a central aspect of the self, such as one’s values, likely confirms for an individual that they know their own attitudes. Thus, beliefs and opinions central to the self (i.e., based in values) may be viewed as “knowable” and therefore clearer than beliefs and opinions that are less value-based and less central to self-concepts. This reasoning is consistent with these attitudes being driven primarily by the motivation to facilitate, clarify, and express values (Katz, 1960; Murray et al., 1996). Moreover, factor analyses of value centrality and attitude knowledge typically load on the same factor (embeddedness; Pomerantz et al., 1995; Prislin, 1996; Philipp-Muller et al., 2020).

This prediction is also bolstered by research on the association between attitude- and self-certainty (e.g., Clarkson et al., 2009). As mentioned previously, repeated expression of an attitude can increase attitude clarity but not correctness (Petrocelli et al., 2007; Cheatham and Tormala, 2015). Clarkson et al. (2009) demonstrated that increasing an attitude’s certainty *via* repeated expression led to increases in self-certainty (one’s clarity of their self-concept), but only for those individuals for whom the attitude reflected core values (i.e., attitude centrality; Pomerantz et al., 1995).

### Research overview

The goals for the current research are two-fold. First, in three studies we examine how the perception that one’s attitudes are based in values can affect attitude certainty—a commonly studied attitude dimension that has important consequences for attitude strength. Building upon previous research on value-attitude relations and attitude certainty (e.g., Maio et al., 2009; Blankenship et al., 2012), we hypothesize that a particular aspect of attitude certainty—attitude clarity—is primarily responsible for the strength-related consequences of having an attitude based in values. Such a finding would indicate that basing an attitude in one’s values may be an antecedent to increased attitude clarity. To this end, we manipulate the perceived value basis of an attitude following exposure to counterattitudinal information. This experimental design offers a stronger test of the hypotheses by establishing causal influences of value basis on the critical dependent measures.

Second, previous research has primarily examined how value-attitude relations influence resistance to persuasion with resistance as an outcome. That is, value-based attitudes lead to changes in favorability (i.e., attitude change), relative to attitudes weakly based in values (Johnson and Eagly, 1990; Blankenship et al., 2012, 2015). In contrast, the present studies examine an appraisal-based framework (Tormala and Petty, 2004; Rucker et al., 2014); attitudes based in values can also affect the attitude dimensions such as certainty, which then led to increases in attitude strength. Specifically, having an attitude based in values can affect the subjective sense that one knows one’s attitude, despite the fact that the attitude does not change (value-issue relations affecting perceptions of resistance). Under these conditions, increases in attitude clarity increase the perception that one has effortfully considered a persuasive attack (Study 2), which then has consequences for perceptions of resistance and intentions to act on the attitude (Study 3).^1^

## Study 1

### Methods

Study 1 manipulated value basis for one’s attitudes. Specifically, following a counterattitudinal message, participants received feedback regarding the extent to which values provide a strong or weak basis of their thoughts about the relevant issue (see Tormala and Petty, 2002 for a similar feedback manipulation). This manipulation was chosen because it is not apparently similar to prior manipulations of attitude clarity and attitude correctness. That is, the manipulation does not seem to differentially enhance either social consensus or repeated expression of an attitude or value.

We selected a contemporary social issue that is normatively pro-attitudinal (civil rights policies) and that has a relatively intuitive association with various values. Pretesting (*n* = 182) revealed overall favorable attitudes toward civil rights policies (*M* = 6.97, *SD* = 1.51; 1 = *definitely opposed*; 9 = *definitely in favor*). Moreover, similar to previous research (Rokeach, 1973; Schwartz, 1994, 2006), the pretesting revealed that the combined importance ratings of the values equality, social justice, and broadmindedness (*M* = 5.75, *SD* = 0.83; 1 = *not at all important*; 7 = *very important; α = 0.59*) were correlated with favorability of civil rights policies even when controlling for the average importance rating of all values in the pretest, *r*(181) = 0.25, *p* = 0.001.

### Participants and design

Sample size for Study 1 was determined by the number of participants we anticipated recruiting from the start of the study until the end of the academic semester. We anticipated the final sample would contain at least 60 participants per group, which is consistent with previous research that has used a similar false feedback manipulation (e.g., Tormala and Petty, 2002, Luttrell et al., 2016). The results of a sensitivity analysis revealed that a sample size of 180 and three groups could detect a moderate effect size of *f* = 0.23. As a result, 190 undergraduate students (118 female, 69 male; *M*_*age*_ = 18.96, *SD*_*age*_ = 1.44; 93% Caucasian) participated in a between-participants design with three groups.^2^

### Procedure

Participants were brought into a lab and seated at a computer where all measures and manipulations were administered. Participants were told that they would be participating in a study on reading skills. Participants first read an editorial (ostensibly published in a journal) advocating the elimination of civil rights policies. The message contained four arguments outlining how civil rights policies have been detrimental to society. Specifically, the message provided fictional information that (a) many members of underprivileged groups fall into the middle or upper class, (b) civil rights policies lower the level of accountability by underprivileged groups, (c) lowering academic standards results in students from underprivileged backgrounds being unable to keep up, and (d) it is condescending and insulting to imply that members of underprivileged groups cannot achieve their goals through hard work and ability.

After reading, participants were instructed to write thoughts about the editorial. Participants in the feedback conditions were then provided bogus feedback concerning the extent to which their thoughts were based in their important values. Specifically, using a paradigm by Tormala and Petty (2002), we told participants the computer would analyze the extent to which their thoughts originated from their important values. Following the feedback, participants reported their attitudes toward civil rights policies and then completed the attitude clarity and correctness measures. Participants in the control condition did not receive any feedback; they completed the dependent measures after writing their thoughts. Afterward, participants were debriefed about the fictitious nature of the feedback and the purpose of the research.

### Independent variable

#### Value basis induction

After reading the editorial and reporting their thoughts, participants were randomly assigned to one of three feedback conditions. In the No Feedback (i.e., control) condition, participants did not receive any feedback about their thoughts. This condition provided a baseline for attitude favorability and attitude clarity and correctness. In the other two feedback conditions, participants were told that the computer would analyze the extent to which their important values served as a basis for their thoughts and calculate a Value Basis Index. The index could range from 1 to 10, with higher scores indicating their values serve as a strong basis for their thoughts. After a 5-s delay used to simulate the basis calculation, participants in the Weak Feedback condition were told their thoughts were weakly based on their important values (2 out of 10), whereas participants in the Strong Feedback condition were told that their thoughts were strongly based in their important values (9 out of 10).

### Dependent variables

#### Thoughts

Directly after reading the editorial, participants were asked to list up to six thoughts they had while reading the message. Overall, participants generated an average of 3.76 thoughts (*SD* = 1.53). After reporting their thoughts, participants rated each of their own thoughts in terms of its favorability toward the message. That is, participants rated whether each thought was in favor of civil rights policies (coded as +1), against civil rights policies (coded as −1), or neutral/irrelevant toward civil rights policies (coded as 0). Thought favorability was calculated by subtracting the number of thoughts against the policies from the thoughts favoring the policies, divided by the total number of thoughts (see Wegener et al., 1995). Negative numbers suggest greater opposition to the editorial against civil rights policies.

#### Attitudes

Following the Value Basis manipulation, participants reported their attitudes toward civil right policies on six 9-point scales (1 = *bad, strongly disagree, foolish, harmful, unfavorable, and definitely do not approve*; 9 = *good, strongly agree, wise, beneficial, favorable, and definitely approve*, respectively; α = 0.96).

#### Attitude clarity and correctness

After reporting their attitudes, participants completed four attitude clarity and three attitude correctness items adapted by Petrocelli et al. (2007); see also Cheatham and Tormala, 2015) on 9-point scales (1 = *not certain at all*, 9 = *very certain*). Items were presented randomly; both clarity (α = 0.93) and correctness (α = 0.83) demonstrated adequate reliability.

#### Value basis manipulation check

After reporting their attitudes and attitude clarity, all participants reported their value basis index they received earlier on a 10-point scale.

### Results

Table 1 reports the means and standard deviations for the variables of interest.

**TABLE 1:** Study 1 means and standard deviations for the relevant dependent measures as a function of the value basis condition.

Dependent measure	Control	Weak basis	Strong basis
	*M (SD)*	*M (SD)*	*M (SD)*
Manipulation check	−	2.13 (0.72)_a_	8.95 (0.37)_b_
Thought favorability	−0.09 (0.19)_a_	−0.03 (0.2)_a_	−0.04 (0.18)_a_
Attitudes	5.45 (1.64)_a_	5.85 (1.53)_a_	5.64 (1.55)_a_
Attitude clarity	5.59 (1.87)_a_	5.57 (1.73)_a_	6.36 (1.73)_b_
Attitude correctness	4.92 (1.69)_a_	5.18 (1.69)_a_	5.57 (1.55)_a_

Interpret subscripts within rows.

Means with the same subscript do not differ from each other. Means with different subscripts differ at *p* < 0.05.

#### Manipulation check

To examine whether participants attended to the value basis feedback, we conducted a one-way analysis of variance (ANOVA) on the value basis index they reported. Participants in the Strong Feedback basis condition reported a higher value basis index than participants in the Weak Feedback basis condition *F*(1, 124) = 4556.38, *p* < 0.001. Thus, participants attended to the bogus feedback, increasing the possibility that the manipulation would create perceived differences in value basis and affect the dependent variables of interest.

#### Thought favorability

Because the value basis manipulation occurred after participants reported their thoughts, we expected and found that thought favorability did not differ across the value basis conditions *F*(2, 184) = 1.71, *p* = 0.18 (see Table 1). Therefore, any effect on attitudes and the facets of attitude certainty could not be accounted for by any differences in thought favorability.

#### Attitudes

A one-way ANOVA revealed no omnibus effect of Value Basis *F*(2, 187) = 1.0, *p* = 0.36 (see Table 1). Attitudes did not differ across the feedback conditions, suggesting that the feedback manipulation did not affect attitudes. Moreover, any change in attitude clarity and correctness as a function of the Value Basis manipulation is likely not a function of change in attitudes.

#### Attitude clarity and correctness

We conducted a similar analysis on the attitude clarity and correctness measures. A significant effect of Value Basis emerged *F*(2, 187) = 4.11, *p* = 0.02, η^2^_*p*_ = 0.04 (see Table 1). As expected, orthogonal contrasts revealed that participants in the Strong Feedback condition reported greater attitude clarity than participants in both the Weak Feedback *F*(1, 187) = 6.12, *p* = 0.01, η^2^_*p*_ = 0.05 and No Feedback (control) conditions, *F*(1, 187), = 6.08, *p* = 0.01, η^2^_*p*_ = 0.04, which did not differ from each other, *F*(1, 187) = 0.002, *p* = 0.96. However, the same analysis on the attitude correctness measure did not yield a significant omnibus effect *F*(2, 187) = 2.53, *p* = 0.08, suggesting that attitude correctness was not affected by the value basis manipulation.

Similar to previous research (e.g., Petrocelli et al., 2007) attitude clarity and correctness were correlated (*r* = −0.70, *p* < 0.001). We therefore conducted an analysis of covariance (ANCOVA) examining the effect of Value Basis on attitude clarity while controlling for attitude correctness. Results revealed that the effect on clarity remained significant *F*(1, 123) = 5.0, *p* = 0.03, η^2^_*p*_ = 0.04, after controlling for attitude correctness, suggesting that the value basis manipulation affected attitude clarity while controlling for attitude correctness. In addition, an ANCOVA with attitude correctness as the dependent measure and attitude clarity as a covariate revealed no significant effect of Value Basis *F*(1, 123) = 0.43, *p* = 0.50.

### Discussion

Participants who learned their thoughts on a counterattitudinal message were strongly based in values reported increases in attitude clarity to a greater extent than attitude correctness, when compared to participants who received opposite feedback. Furthermore, comparisons with the no-feedback control condition revealed that the manipulation did not affect attitude favorability.

We thus sought to replicate the effects and extend them in Study 2. We modified the paradigm in three ways. First, the value basis feedback used in Study 1 was rather general and did not indicate the specific type of value relevant to participants’ thoughts. It is therefore unclear whether the perception that an attitude is based on any value may increase attitude clarity, or if the type of value matters. In other words, one’s values could be of low relevance to, or even incompatible with, the topic and yet provide support for the topic. Simply attributing a participant’s thoughts to any positive cognitive quality such as a value would enhance the strength of an attitude (Blankenship and Wegener, 2008).

However, it should be noted that in the present research we used civil rights policies, a topic that has direct relevance to universalism values (e.g., equality, social justice, etc.; see also footnote 3). Moreover, some motivations driving various value types can be incompatible (e.g., universalism vs. achievement; Schwartz, 1992, Pakizeh et al., 2007; Maio et al., 2014). Thus, perceived support by values incompatible with the topic may not affect the subcomponents of attitude certainty, suggesting that simply attributing a participant’s thoughts to any value may not enhance the strength of an attitude. Therefore, in Study 2, we modified the bogus feedback such that all participants were told that their attitudes are based in values, but the compatibility of the values with the topic varied. Some individuals were told that their attitude was based in universalism values, whereas others were told that their attitude was based in achievement values.

Because the specificity of the value was manipulated, this paradigm also afforded us the opportunity to examine how individual differences in one’s importance for universalism over achievement moderate the hypothesized effect. We therefore included a measure of value importance to examine this possibility. We hypothesized that participants who were told that their thoughts are based in the universalism values would have a greater influence on attitude clarity than attitude correctness, particularly for those who report a greater importance preference for universalism than achievement values.

Finally, we were also interested in examining consequences of attitude clarity appraisals on elaboration. Because increases in attitude certainty are associated with increased perceptions that one has processed attitude-relevant information (Barden and Petty, 2008) we added a measure of subjective elaboration.

## Study 2

### Methods

#### Participants and design

We used the effect size from Study 1 for the effect of value basis feedback on attitude clarity (*d* = 0.45) to inform sample size for Study 2. Ninety-seven undergraduate students (53 female, 44 male; *M*_*age*_ = 19.28, *SD*_*age*_ = 2.06; 88% Caucasian) participated in a 2(Value Type: Achievement vs. Universalism) × Relative Value Importance (continuous) design.

### Procedure

Participants were told that they would be participating in two separate studies, the first being a general opinion survey and the second a survey of reading skills. Participants first rated the importance of 25 values adapted from Schwartz (1992). Participants were then exposed to the reading skills instructions and anti-civil rights message used in Study 1. The remaining procedures were the same as Study 1 with two exceptions. First, we omitted the No Feedback control condition; all participants received (bogus) feedback after writing their thoughts about the editorial. Second, all participants were told that their thoughts were strongly based in values, but the specific values differed across conditions. Specifically, some participants were informed that their thoughts were based in universalism values (e.g., equality), whereas others were told that their thoughts were based in achievement values (e.g., ambition).

### Independent variables

#### Value importance

At the beginning of the session, participants reported the importance of 25 values or guiding principles in their lives on separate 7-point scales (1 = *Not at all important to me*; 7 = *Very important to me*). The values of interest were three achievement-related values (ambition, social power, wealth) and three universalism values (broadmindedness, equality, social justice). The achievement-related values were combined to create a single index of achievement-related value importance (*M* = 4.78, *SD* = 0.82; α = 0.40). Similarly, the universalism-related values were combined to create a single index of universalism-related value importance (*M* = 5.81, *SD* = 0.92; α = 0.69). We created a Relative Value Importance index by subtracting the aggregated score of achievement values from the aggregated score of universalism values. Positive scores represent greater importance for universalism over achievement.^3^

#### Thought feedback

Similar to Study 1, participants were given bogus feedback regarding which values served as a basis for their thoughts. However, participants in the achievement condition were told that ambition, social power, and wealth (i.e., the achievement-related values) served as the basis for their thoughts, whereas participants in the universalism condition were told that broadmindedness, equality, and social justice (i.e., the universalism-related values) served as the basis for their thoughts.

### Dependent variables

#### Thoughts

As with Study 1, participants could list up to six thoughts; thought favorability was calculated with the same procedures. Participants generated an average of 3.35 thoughts (*SD* = 1.46). The mean favorability was generally negative, opposing the editorial’s position on ending civil rights policies (*M* = −0.13, *SD* = 0.61).

#### Attitudes

After writing their thoughts, participants reported their attitudes toward civil right policies on the same six 9-point scales used in Study 1 (α = 0.97).

#### Attitude clarity and correctness

Following the attitude measure, participants completed the same attitude clarity (α = 0.93) and correctness (α = 0.82) measures used in Study 2. The two measures were correlated (*r* = 0.69).

#### Self-reported elaboration

Following attitude clarity and correctness, we assessed perceived elaboration on four 9-point scales. Specifically, participants reported how much attention they paid to the message (1 = *no attention at all*; 9 = *a lot of attention*), how deeply they thought about the message (1 = *not deeply at all*; 9 = *very deeply*), how much effort they put into reading the message (1 = *no effort at all*; 9 = *a lot of effort*), and how personally involved they felt with the topic (1 = *not involved at all*; 9 = *very involved*). Responses were combined to create a single index of perceived elaboration (α = 0.83; see also Tormala et al., 2006).

### Results

#### Thoughts

We conducted a 2(Value Type: Achievement vs. Universalism) × Relative Value Importance (continuous; centered) moderated regression on participants’ thought favorability using the PROCESS macro for SPSS (Model 1; Hayes, 2022). Results revealed neither Value Type (*p* = 0.17), Relative Value Importance (*p* = 0.08) nor their interaction on thought favorability (*p* = 0.90) reached traditional significance. Thus, any effect on attitude certainty and perceived elaboration could not be accounted for by differences in thought favorability toward the civil rights issue.

#### Attitudes

Moderated regression analysis on participants’ attitudes revealed that participants with greater relative importance for universalism reported more favorable attitudes toward civil rights policies, *b* = 0.32, *SE* = 0.11, *t*(93) = 2.91, *p* = 0.005, 95% *CI*: [0.1, 0.54]. No other effects were significant (*p*s > 0.27). Thus, as in Study 1, attitudes toward civil rights policies did not differ across the feedback conditions.

#### Attitude clarity and correctness

**FIGURE 1:**
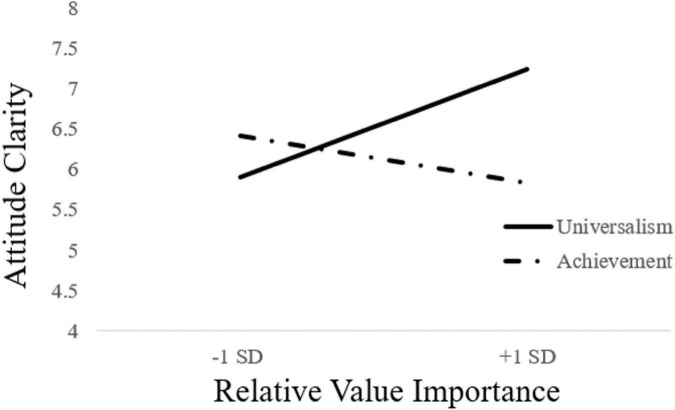
Study 2 value type × relative value importance effect on attitude clarity.

We expected that participants who rate universalism as being relatively more important than achievement (as indicated by positive scores on the relative importance index) and who were told that their thoughts were based in universalism, would report greater attitude clarity than participants who were told their thoughts were based in achievement. A moderated regression analysis with attitude clarity as the dependent measure revealed the predicted interaction, *b* = 0.31, *SE* = 0.12, *t*(93) = 2.66, *p* = 0.009, 95% *CI*: [0.08, 0.53]. Decomposition of the interaction revealed that at 1 *SD* above the mean of Relative Value Importance, participants in the Universalism feedback condition reported greater attitude clarity (*M* = 7.3) than participants in the Achievement feedback condition (*M* = 5.85), *b* = 0.72, *SE* = 0.26, *t*(93) = 2.75, *p* = 0.007, 95% *CI*: [0.2, 1.24] (see Figure 1). For participants 1 *SD* below the mean of Relative Value Importance, there was no significant difference on attitude clarity between the Universalism feedback condition (*M* = 5.90) and the Achievement feedback condition (*M* = 6.43), *b* = −0.27, *SE* = 0.26, *t*(93) = −1.01, *p* = 0.31, 95% *CI*: [−0.79, 0.25]. Thus, participants for whom universalism is more important than achievement reported greater attitude clarity when provided feedback that their thoughts were based in universalism rather than achievement.

A similar moderated regression analysis on the attitude correctness measure revealed a marginal effect of Value Type on attitude correctness *b* = 0.29, *SE* = 0.17, *t*(93) = 1.71, *p* = 0.09, 95% *CI*: [−0.05, 0.64], with participants in the Universalism feedback condition reporting greater attitude correctness (*M* = 5.78) than in the Achievement feedback condition (*M* = 5.19). Other effects were not significant, including the interaction, *b* = 0.15, *SE* = 0.11, *t*(93) = 1.37, *p* = 0.17, 95% *CI*: [−0.07, 0.36]. Thus, the match between the Value Type feedback and participants’ relative importance affected attitude clarity to a greater degree than attitude correctness.

Due to the strong correlation between attitude clarity and attitude correctness, a moderated regression analysis with attitude correctness as a covariate on the attitude clarity measure revealed that, even when controlling for attitude correctness, the interaction between Value Type and value importance remained significant *b* = 0.20, *SE* = 0.08, *t*(92) = 2.32, *p* = 0.02, 95% *CI*: = [0.03, 0.37]. This suggests that the Value Type × Relative Value Importance interaction held while controlling for attitude correctness.

We conducted similar analyses with the ipsative transformation of the importance scores as a predictor, calculating the average importance of all 25 values and subtracting this from the average importance of achievement and universalism. The results did not change appreciably and remained significant.

#### Self-reported elaboration

**FIGURE 2:**
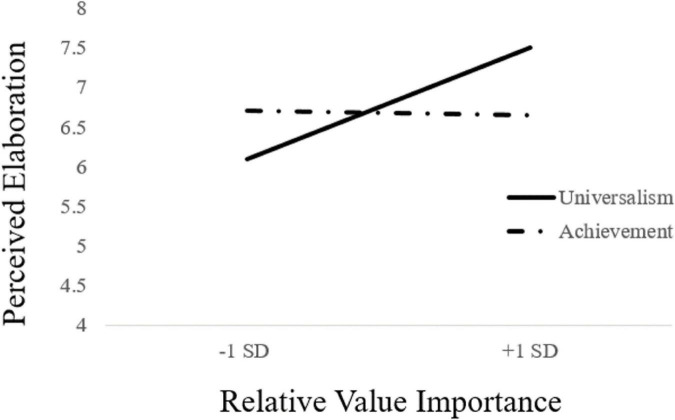
Study 2 value type × relative value importance effect on perceived elaboration.

We expected a similar pattern on the perceived elaboration measure as the attitude clarity measure. Results of a 2(Value Type: Achievement vs. Universalism) × Relative Value Importance (continuous; centered) moderated regression revealed that participants with greater relative importance for universalism reported greater perceived elaboration, *b* = 0.22, *SE* = 0.1, *t*(93) = 2.11, *p* = 0.04, 95% *CI*: [0.01, 0.43]. More importantly, a significant interaction emerged, *b* = 0.26, *SE* = 0.10, *t*(93) = 2.48, *p* = 0.02, 95% *CI*: [0.05, 0.47]. Decomposition of the interaction in the same manner as with the above analyses revealed that, for participants 1 *SD* above the mean of participants’ value difference scores, participants in the Universalism feedback condition reported elaborating on the message (*M* = 7.63) more than participants in the Achievement feedback condition (*M* = 6.66), *b* = 0.48, *SE* = 0.24, *t*(93) = 2.03, *p* = 0.05, 95% *CI*: [0.01, 0.96] (see Figure 2). For participants 1 *SD* below the mean of participants’ value difference scores, there was no significant difference in self-reported elaboration between participants in the Universalism feedback condition (*M* = 6.07) and those in the Achievement feedback condition (*M* = 6.78), *b* = −0.35, *SE* = 0.24, *t*(93) = −1.48, *p* = 0.14, 95% *CI*: [−0.83, 0.12]. Put differently, participants whose relative value importance favored universalism over achievement reported greater elaboration of the message when told that their thoughts were based in universalism.

### Mediation analyses

#### Perceived elaboration as mediator on attitude clarity

As noted, the Value Type × Relative Value Importance interaction affected attitude clarity and perceived elaboration. We thus examined whether, consistent with previous research (e.g., Barden and Petty, 2008), increases in perceived elaboration would lead to increases in attitude certainty. Specifically, we expected that differences in perceived elaboration would mediate the effect of bogus feedback on attitude clarity for participants who rate universalism as more important than achievement. To test this, we conducted a moderated mediation analysis using bootstrapping procedures using the PROCESS macro for SPSS (Model 8; Hayes, 2022). The Value Type × Relative Value Importance (centered) term was treated as the distal variable and the perceived elaboration term was treated as a potential mediator.

**FIGURE 3:**
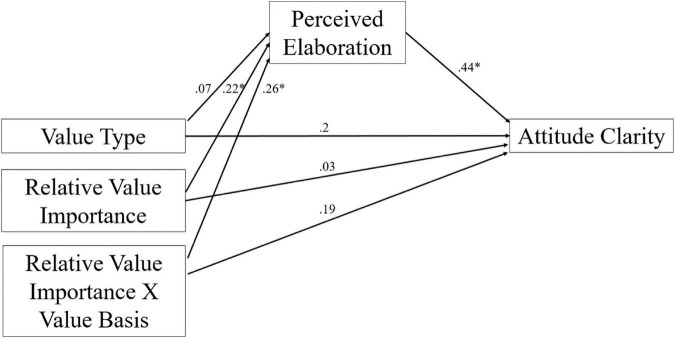
Study 2 moderated mediation of perceived elaboration on value type × relative value importance effects on attitude clarity; *p < 0.05.

Examination of the index of moderated mediation revealed that the higher order indirect effect of perceived elaboration term (*M* = 0.11, *SE* = 0.07) mediated the effect of the Value Type × Relative Value Importance on the attitude clarity measure, 95% BS *CI*: [0.006, 0.26] (see Figure 3). The Value Basis × Relative Value Importance interaction on attitude clarity was no longer significant, *b* = 0.19, *SE* = 0.11, *t*(92) = 1.76, *p* = 0.08, 95% *CI*: [−0.03, 0.41]. Thus, perceived elaboration mediated the effect of the interaction between Value Type and Relative Value Importance on attitude clarity.

We further decomposed the moderated mediation to examine the conditional indirect effects of perceived elaboration at different levels of Relative Value Importance. At 1 *SD* above the mean of Relative Value Importance, the indirect effect (*M* = 0.21, *SE* = 0.11) did not include 0, 95% BS *CI*: [0.02, 0.46], suggesting that perceived elaboration mediated the effect of Value Type on attitude clarity. In other words, participants in the universalism feedback conditions reported increased elaboration and attitude clarity, particularly when they reported greater importance for universalism values than achievement values. At 1 *SD* below the mean of Relative Value importance, however, the indirect effect (*M* = −0.15, *SE* = 0.14) included 0, 95% BS *CI*: [−0.49, 0.08]. Thus, perceived elaboration mediated the effect of Value Type on attitude clarity for participants with a relative preference of universalism values over achievement values.

We conducted a similar analysis with attitude correctness as a covariate and found that the higher order indirect effect of the perceived elaboration term (*M* = 0.06, *SE* = 0.04) mediated the effect of the Value Type × Relative Value Importance on the attitude clarity measure, 95% BS *CI*: [0.0002, 0.17], even when controlling for attitude correctness. Similarly, the Value Type × Relative Value Importance interaction on attitude clarity was no longer significant, *b* = 0.16, *SE* = 0.09, *t*(91) = 1.82, *p* = 0.07, 95% *CI*: [−0.01, 0.33].

### Discussion

In addition to conceptually replicating Study 1, Study 2 extended the finding that feedback indicating one’s attitudes are based in values increases attitude clarity. That is, despite all participants being told that their attitude was based in values, compatibility between the topic and the value increased attitude clarity. Specifically, the effect occurred for participants who reported the importance of universalism values over achievement values. Such a finding suggests that the compatibility effect matters for those individuals in particular who rate the universalism as more important than the achievement. Thus, participants who place greater importance on universalism than achievement, when told that their thoughts were based in universalism, also reported greater elaboration which was associated with greater attitude clarity.^4^ However, inspection of the value importance ratings indicate that the universalism values were on average rated as more important than the achievement values. Therefore, it may be that value importance in general may be responsible for the effects in Study 2, rather than the relative difference between universalism and achievement values. To test this possibility, we averaged the importance for all twenty-five values in the value inventory and then entered it (centered) in a regression with the Value Type manipulation and their interaction predicting attitude clarity. Results revealed no significant effects (*p*s > 0.23), including the non-significant *b* = 0.38, *SE* = 0.36, *t*(93) = 1.05, *p* = 0.29, 95% *CI*: = [−0.34, 1.1]. Thus, relative value importance *per se* does not moderate the value basis effects on attitude clarity.

## Study 3

Having established the link between value basis and attitude clarity, we conducted Study 3 to conceptually replicate the findings of Studies 1 and 2 on two attitude strength variables: resistance to persuasion and intentions to act on one’s attitude. Previous research on attitude certainty has found that, in addition to affecting subjective elaboration (Barden and Petty, 2008, Study 3) increases in attitude certainty affect the perception that one has resisted a counterattitudinal message (Tormala et al., 2006) and increased intentions to act on an attitude. Importantly, we sought to extend these findings beyond subjective elaboration to the specific subcomponent of attitude clarity. To test this, we reverted to the design and value basis feedback used in Study 1. We also added measures of global certainty, perceived resistance success, and intentions to act on one’s attitude (an indicator of attitude strength).

### Participants and design

Given that the paradigm was similar to Study 1, we used the effects from that study to determine sample size for Study 3. A sensitivity analysis revealed that a sample size of 150 and two groups could detect a moderate effect size of *f* = 0.23. One hundred fifty undergraduate students (95 female, 55 male; *M*_*age*_ = 19.01, *SD*_*age*_ = 1.18; 88% Caucasian) participated in a between-participants design with two groups.

### Procedure

Participants were given the same cover story and procedures from Study 1 with one exception. After reporting their attitudes, participants completed measures of global certainty, attitude clarity and correctness, perceived elaboration, perceived resistance, and a willingness to discuss measure related to their attitudes toward civil rights policies. Afterward, participants were fully debriefed.^5^

### Independent variable

#### Thought feedback

Participants received the same bogus feedback used in Study 1, such that participants were told that their thoughts were either weakly or strongly based in their important values.

### Dependent variables

#### Thoughts

Participants listed up to six thoughts and thought favorability was calculated the same way as in Studies 1 and 2. Participants generated an average of *M* = 3.72 thoughts (*SD* = 1.36).

#### Attitudes

Following the feedback manipulation, participants reported their attitudes toward civil right policies on the same six 9-point scales used in Study 1 (α = 0.94).

#### Global certainty

Following the attitude measure, participants completed two global certainty items on 9-point scales. Specifically, participants reported the extent to which they are certain of their attitudes (1 = *not certain at all*; 9 = *very certain*) and how much confidence they have in their attitudes (1 = *no confidence at all*; 9 = *a great deal of confidence*). Reponses to the items were correlated (*r* = 0.87, *p* < 0.001) and were therefore combined to create a global measure of attitude certainty (see Petrocelli et al., 2007, for a similar measure).

#### Attitude clarity and correctness

After reporting their global certainty, participants completed the same attitude clarity (α = 0.95) and attitude correctness (α = 0.87) measures used in Studies 1 and 2.

#### Perceived success resisting

After the certainty measures, participants reported how well they resisted the editorial on three 9-point scales. Specifically, participants reported the extent to which their thoughts were strong (1 = *not very strong*; 9 = *very strong*), how effective they were at maintaining their initial attitude toward civil rights policies (1 = *not very effective*; 9 = *very effective*), and how successful they were in resisting the information. Reponses to the items were related (α = 0.75) and were therefore combined to create a global measure of resistance success (see Tormala and Petty, 2002, for a similar measure).

#### Self-reported elaboration

Participants completed the same perceived elaboration items used in Study 2 (α = 0.84).

#### Intentions

After reporting perceived elaboration, participants reported their willingness to discuss their attitude toward civil rights policies (a) with someone who has an opposing viewpoint, (b) in public, and (c) in a petition that supports their attitude toward civil rights policies (1 = *not at all willing*; 9 = *very willing*.) These were correlated (α = 0.85) and were therefore combined to create a global measure of intentions to act.

#### Manipulation check

After reporting their attitudes, participants completed the value basis index from Study 2.

### Results

Table 2 reports the means and standard deviations for the variables of interest.

**TABLE 2:** Study 3 means, standard deviations, and effect sizes for thought favorability, attitudes, attitude clarity, and attitude correctness as a function of the feedback manipulation.

Dependent measure	Weak basis	Strong basis	*F*	*p*	*d*
	*M (SD)*	*M (SD)*			
Manipulation check	2.07 (0.48)	8.99 (0.12)	15054.92	<0.001	20.17
Thought favorability	−0.02(0.22)	0.02 (0.23)	1.82	0.18	0.22
Attitudes	5.82 (1.42)	6.0 (1.42)	0.65	0.42	0.13
Global certainty	5.59 (1.79)	6.2 (1.78)	4.55	0.04*	0.35
Attitude clarity	5.64 (1.89)	6.57 (1.73)	9.89	0.002**	0.52
Attitude correctness	5.2 (1.56)	5.64 (1.66)	2.86	0.09	0.28
Perceived elaboration	6.15 (1.47)	6.63 (1.23)	4.68	0.03*	0.36
Perceived resistance	5.09 (1.59)	6.02 (1.38)	14.8	< 0.001***	0.63
Intentions	4.88 (2.11)	5.62 (2.09)	4.69	0.03*	0.36

*p < 0.05, **p < 0.01, ***p < 0.001.

#### Manipulation check

A one-way ANOVA on the value basis index revealed that, similar to Study 1, participants in the strong basis condition reported a higher value basis index than participants in the weak basis condition, *F*(1, 148) = 15054.92, *p* < 0.001, η^2^_*p*_ = 0.99. Meaning, participants attended to the feedback, therefore increasing the opportunity that the manipulation would affect the dependent variables of interest.

#### Thoughts

A similar analysis on the thought favorability measure revealed that thought favorability did not differ across the value basis conditions *F*(1, 148) = 1.82, *p* = 0.18.

#### Attitudes

Similar to Studies 1 and 2, attitudes did not differ across the value basis conditions *F*(1, 148) = 0.65, *p* = 0.42.

#### Attitude certainty

A one-way ANOVA on the attitude certainty measures revealed that participants in the strong feedback conditions reported greater global certainty *F*(1, 148) = 4.55, *p* = 0.04, η^2^_*p*_ = 0.03 and attitude clarity *F*(1, 148) = 9.89, *p* = 0.002, η^2^_*p*_ = 0.06 than those in the weak feedback conditions. The difference in attitude correctness between conditions was not significant *F*(1, 148) = 2.86, *p* = 0.09. Consistent with Studies 1 and 2, the value basis manipulation affects perceptions of attitude clarity to a greater extent than attitude correctness.

#### Self-reported elaboration

A one-way ANOVA on the self-reported elaboration measure revealed that participants in the strong value basis condition reported greater elaboration than in the weak value basis condition *F*(1, 148) = 4.68, *p* = 0.03, η^2^_*p*_ = 0.03.

#### Perceived success resisting

A one-way ANOVA on the resistance success measure revealed that participants in the strong value basis conditions reported greater success resisting *F*(1, 148) = 14.80, *p* < 0.001, η^2^_*p*_ = 0.09 than in the weak value basis conditions.

#### Intentions

A one-way ANOVA on the intentions measure revealed that participants in the strong value basis condition reporting greater intentions to act on their attitude than in the weak value basis condition *F*(1, 148) = 4.70, *p* = 0.03, η^2^_*p*_ = 0.03.

### Mediation analyses

#### Subjective elaboration mediation on attitude clarity

We examined the conceptual replication of the mediation effects in Study 2 wherein perceived elaboration mediated the effect of the value basis manipulation on attitude clarity (Barden and Petty, 2008). We conducted a mediation analysis using the PROCESS macro for SPSS (Model 4; Hayes, 2022). The 95% confidence interval on the indirect effect suggested that the indirect path through perceived elaboration mediated the effect of value basis (coded as weak = −1; strong = +1) on attitude clarity (*b* = 0.13, *SE* = 0.07, 95% *CI*: 0.01, 0.30). The direct effect of the value basis manipulation remained significant, but was reduced [*b* = 0.33, *SE* = 0.14, *t*(147) = 2.41, *p* = 0.02, 95% *CI*: 0.06, 0.6]. Thus, consistent with Study 2, feedback that one’s thoughts were strongly based in one’s values was associated with greater perceived elaboration and with greater attitude clarity.

#### Attitude clarity mediation on resistance success

We examined whether, consistent with Tormala and Petty (2002), attitude clarity would mediate the effect of value basis on perceived success resisting. The 95% confidence interval on the indirect effect suggested that the indirect path through attitude clarity mediated the effect of value basis (coded as weak = −1; strong = +1) on perceived resistance success (*b* = 0.24, *SE* = 0.08, 95% *CI*: 0.09, 0.41). The direct effect of the value basis manipulation remained significant, but was reduced [*b* = 0.23, *SE* = 0.1, *t*(147) = 2.32, *p* = 0.03, 95% *CI*: 0.06, 0.60].

#### Attitude clarity mediation on intentions

We examined whether attitude clarity would mediate the effect of value basis on intentions. The 95% confidence interval on the indirect effect suggested that the indirect path through attitude clarity mediated the effect of value basis (coded as weak = −1; strong = +1) on intentions (*b* = 0.24, *SE* = 0.09, 95% *CI*: 0.09, 0.44). The direct effect of the value basis manipulation was no longer significant [*b* = 0.12, *SE* = 0.16, *t*(147) = 0.79, *p* = 0.43, 95% *CI*: −0.19, 0.46].

#### Serial mediation results

As noted in the previous sections, participants who were told their thoughts were based on values had greater subsequent attitude clarity, greater perceived elaboration, greater perceived resistance to change, and greater willingness to act on the attitude. Because perceived elaboration mediates effects of distal variables on attitude certainty (Tormala et al., 2006; Barden and Petty, 2008) and attitude strength (Barden and Petty, 2008), it may be that perceived elaboration and attitude clarity represent sequential points in the causal progression to influencing perceived resistance and intentions. To test these possibilities, we conducted separate multiple step mediation models using bootstrapping procedures outlined by Hayes et al. (2010) using the PROCESS macro for SPSS (Model 6; Hayes, 2022).

#### Perceived elaboration and attitude clarity mediation on resistance success

**FIGURE 4:**
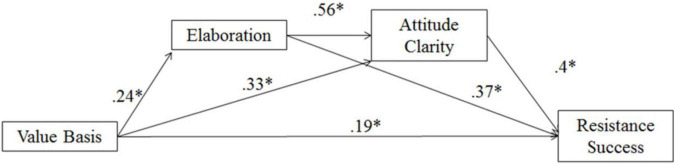
Multiple mediation of value basis on resistance success with perceived elaboration and attitude clarity as mediators in Study 3; *p < 0.05.

The first model examined perceived resistance as an outcome variable. As in Study 2, the bootstrapping analyses randomly drew cases from the sample data (with replacement) and created 5000 bootstrap data sets of equal size to the original sample. Each data set supplied an estimate of the indirect (mediational) effect of perceived elaboration and attitude clarity as potential mediators (separately and with perceived elaboration as M_1_ and attitude clarity as M_2_) on perceived resistance. The 95% confidence interval on the indirect effects suggested that the indirect path through perceived elaboration and attitude clarity mediated the effect of value basis (coded as weak = −1; strong = +1) on perceived resistance (*b* = 0.05, *SE* = 0.03, 95% *CI*: 0.009, 0.13; see Figure 4). With the mediators in the model, the direct effect of the value basis manipulation remained significant, but reduced [*b* = 0.19, *SE* = 0.10, *t*(146) = 2.02, *p* = 0.04, 95% *CI*: 0.01, 0.38]. In other words, the findings are consistent with the hypothesis that feedback that one’s thoughts were strongly based in one’s values was associated with a greater perceived elaboration, which was associated with greater attitude clarity, which then increased perceptions that one resisted the message.

#### Perceived elaboration and attitude clarity mediation on intentions

**FIGURE 5:**
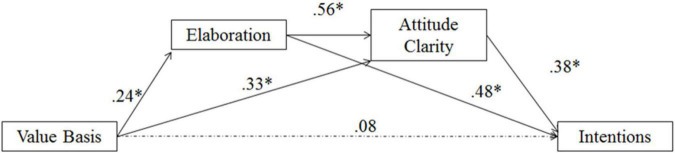
Multiple mediation of value basis on intentions with perceived elaboration and attitude clarity as mediators in Study 3; *p < 0.05.

The second model examined intentions to act on the attitude as the outcome variable. Using the same methods outlined above, we examined the indirect (mediational) effect of perceived elaboration and attitude clarity as potential mediators in the same fashion as in the first model on intentions. The 95% confidence interval on the indirect effects suggested that the indirect path through perceived elaboration and attitude clarity mediated the effect of value basis (coded as weak = −1; strong = +1) on intentions (*b* = 0.05, *SE* = 0.03, 95% *CI*: 0.01 to 0.14; see Figure 5). The direct effect of the value basis manipulation was no longer significant [*b* = 0.08, *SE* = 0.15, *t*(146) = 0.51, *p* = 0.61, 95% *CI*: −0.23, 0.39]. Thus, similar to resistance success as an outcome, feedback that one’s thoughts were strongly based in one’s values was associated with a greater perception that one thought effortfully about the message, which was associated with greater attitude clarity, which was related to increased intentions to act on the attitude.^6^

## General discussion

Previous research has demonstrated that an attitude’s association with values is an important consideration in determining an attitude’s strength (Rokeach, 1968). Attitude certainty plays a meaningful role in imparting strength to a value-relevant attitude (Blankenship et al., 2012). These three studies build upon previous research by demonstrating that learning one’s attitude is value-based primarily affects attitude clarity, a specific subcomponent of attitude certainty. This increase in clarity is in part associated with increased perceptions that one has deeply processed counterattitudinal information (Studies 2 and 3). Attitude clarity, in turn, was associated with the perception that one has successfully resisted the attack on one’s attitude and increases intentions to act on the attitude (Study 3). In other words, having a value-based attitude predicts the sense that one truly knows one’s attitude, which then imparts strength on the attitude. Attitude correctness, on the other hand, seems to not be as consequential in affecting such strength-related appraisals.

The current studies make a number of contributions to the psychological literature on value-attitude relations and persuasion. First, the studies extend knowledge about the mechanism for value-attitude effects on persuasion by demonstrating that a subcomponent of certainty, attitude clarity, is an important mechanism for an attitude’s increased strength following a weak persuasive attempt. This reasoning is consistent with a metacognitive perspective on value-attitude relations (Maio et al., 2009; Blankenship et al., 2012), which has previously demonstrated that attitude certainty is an important consideration in interattitudinal structure. The present studies also demonstrate the utility of an appraisal-based perspective on values and attitude change; while attitude favorability may not change following an attack, perceptions of certainty, subjective elaboration, and resistance may be bolstered by an attitude’s association with values.

The current studies also provide a novel way of manipulating value basis or value expression. Previous studies have relied on correlational designs where participants are asked to report how much an attitude expresses a particular value (Kristiansen and Zanna, 1991), how relevant a value is to an attitude (Kristiansen and Matheson, 1990; Pomerantz et al., 1995) or how a value relates to the self (Clarkson et al., 2009). While a few manipulations of value-attitude relevance exist, these have made salient multiple attitude functions beyond value expression (Murray et al., 1996), and are specific to a particular set of values (e.g., Maio and Olson, 1995). The present studies adapted a bogus feedback manipulation that does not appear to change other properties of the attitude (favorability, extremity) while also successfully manipulating perceptions about the value basis for attitudes. Additionally, the bogus feedback manipulation is relatively flexible and can be used to examine general effects of value basis on certainty (as in Studies 1 and 3) and effects of specific values on certainty (as in Study 2). It should be noted, however, that while participants in general were able to successfully recall their value basis index, it is unclear whether they fully internalized the feedback. It would be useful for future research to include a measure that would assess participants’ ability to internalize the feedback as an additional manipulation check.

The current studies also contribute to our understanding of attitude certainty. Subcomponents of attitude certainty outlined different antecedents to attitude clarity and correctness. The current research examines a new antecedent of attitude clarity (Petrocelli et al., 2007).^7^ The link between one’s attitudes and values can increase an attitude’s resistance similar to the effect of repeated expression of an attitude. Thus, the sense of knowing one’s attitude may increase through repeated expression and through the perception that one’s attitude is based in values. Given the nature of the two types of manipulations, it is unlikely that the two sources of attitude clarity are redundant; they may therefore increase clarity through different mechanisms. Repeated expression affects the perceived ease with which an attitude comes to mind (Petrocelli et al., 2007). Basing an attitude in values, on the other hand, may increase these sense that the attitude is linked to the self, thus increasing clarity. Future research should examine these possibilities.

### Directions for future research

Despite the advances outlined above, a number of questions remain for future research. For example, while the effects of value bases on attitude clarity are consistent across the studies, it may be possible for an attitude’s association with important values to enhance attitude correctness to a greater extent than attitude clarity under certain conditions. There is also little reason to believe that the values used in Study 2 are consistent with knowing one’s attitude beyond any other commonly studied value.

Broadly, we believe that an attitude’s association with values will be more likely to affect attitude clarity than correctness (i.e., a main effect prediction). However, given the multifaceted nature of the values construct (Rohan, 2000), we submit that it may be possible to create a context where basing an attitude in values using a social consensus paradigm (e.g., learning that one’s attitude is supported by values are similar to a majority of one’s peers) or priming values that do promote social-affiliative motivations may lead values to enhance attitude correctness over clarity. It may even be that attitudes based in values that are linked to affiliation or consensus are viewed as more correct than clear. For example, the universal values of belongingness and social recognition outlined by Schwartz (1992) may be candidates (see also Baumeister and Leary, 1995) to affect perceived attitude correctness rather than attitude clarity. Alternatively, we believe there may even be values tied to knowing one’s self that may drive perceptions of attitude clarity over correctness. For example, the value of inner harmony (cf. Schwartz, 1992) might have motivations tied to attitude clarity more than correctness. These value-priming types of possibilities may be most likely when the attitude’s relation to the value is relatively ambiguous (Higgins et al., 1977).

The current research also has implications for self-monitoring processes in persuasion. High self-monitors, whose attitudes are often driven by concerns with appearances, tend to be more persuaded by persuasive appeals that make information related to image maintenance salient than information relevant to product quality (DeBono and Harnish, 1988). Conversely, low self-monitors, whose attitudes tend to be less prone to image concerns, tend to be more persuaded by message quality and source expertise in persuasive appeals (see also Evans and Clark, 2012). Research from a matching perspective has found that these effects occur in part because the match between the motivation and the information in the persuasion context increases processing of the information relevant to self-monitoring tendencies (i.e., high self-monitors process image-relevant information more than message relevant information, and vice versa for low self-monitors; Petty and Wegener, 1998). This match between the self-monitoring tendency and the information also increase certainty (Evans and Clark, 2012). To date, however, no research has examined the differences in the subcomponents of attitude certainty as a function of the match. Specifically, high self-monitors, because of their frequent processing of social cues (Snyder, 1974) may come away from a persuasive context feeling that their attitude is more correct than clear when the appeal highlights image-relevant information. Conversely, low self-monitors, who tend to process appeals relevant to information quality may report increased attitude clarity but not correctness after processing a quality-relevant appeal.

By separating attitude clarity and correctness, future research may be able to outline the types of specific behaviors one may engage in when an attitude is based in values. For example, attitude clarity is associated with intentions to share one’s opinion, which seems consistent with the motivation to express values through a particular attitude (Cheatham and Tormala, 2015). These results suggest that, consistent with research on advocacy and intentions, value-expression motivations may be more aligned with intentions to share attitude-relevant information than to persuade others to have a similar opinion (Catapano and Tormala, 2021. For example, one may have a supportive attitude toward civil rights that expresses the value of equality and may therefore engage in behaviors relevant to that expression by sharing one’s opinion or donating time to a particular cause that reflects that attitude. Consistent with this pattern, Maio and Olson (1995) found that attitudes toward donating that were based in values led to increased intentions to donate money (a sharing behavior) beyond subjective norms associated with the attitude, compared to attitudes based in utilitarian function. This type of behavior is in contrast to attitude correctness and its association with intentions to persuade by proselytizing (Cheatham and Tormala, 2015). One final implication for the current research is that simply making salient an attitude’s association with a value may create a durable attitude but may not be enough to motivate people to believe that their attitude is correct. We hope to examine these and other possibilities in future research.

## Data availability statement

The raw data supporting the conclusions of this article will be made available by the authors, without undue reservation.

## Ethics statement

The studies involving human participants were reviewed and approved by Iowa State University. The patients/participants provided their written informed consent to participate in this study.

## Author contributions

KB developed the idea, designed the studies, and wrote the manuscript with feedback and edits from KK and MM. KB conducted and analyzed the studies with feedback from KK and MM. All authors contributed to the article.

## Footnotes

1 No participants were excluded and the studies reported. Differences in degrees of freedom across tests may vary due to missing data.

2 Three participants did not report age or race.

3 In the same pretesting reported in the introduction, the combined importance of the achievement values of ambition, social power, and wealth (*M* = 4.71, *SD* = 0.85; α = 0.43) was not significantly correlated with favorability of civil rights policies when controlling for the importance of twenty-two other values *r*(181) = −0.09, *p* = 0.2. Moreover, when controlling for the overall value importance, universalism values were negatively correlated with achievement values *r*(181) = −0.41, *p* < 0.001.

4 Although there is work that supports the serial pattern of results (Barden and Petty, 2008), given the correlational nature of the measures, we acknowledge that it could also be consistent with other plausible alternative models not tested here (Fiedler et al., 2018).

5 The messages were changed slightly for Study 3 to enhance readability, but the content and main ideas remained the same between the messages used in Studies 1 and 2. For example, in Studies 1 and 2, the preferential treatment information read “When you give preferential treatment to underprivileged groups in admission or hiring practices, you are in effect saying: ‘You are incapable of achieving on your own, so let me help you.’ It is condescending and insulting to imply that members of underprivileged groups cannot achieve their goals through hard work and ability. In Study 3, the information was changed to “When you give preferential treatment to underprivileged groups in admission or hiring practices, you are in effect saying: ‘Your group used to be discriminated against, so let me help you.’ It is condescending and insulting to imply that members of underprivileged groups cannot achieve their goals through hard work and ability.”

6 We also conducted an alternative set of serial mediation models, such that we replaced attitude clarity with attitude correctness. With resistance success as the outcome, results reveal that subjective elaboration mediates the path between the value basis manipulation and resistance success (*b* = 0.1, *SE* = 0.05, 95% *CI*: 0.007, 0.21). The confidence intervals for the other indirect effects included 0. In addition, the direct effect of Value basis on resistance success remains significant (*b* = 0.27, *SE* = 0.09, *t*(147) = 3.01, *p* = 0.003 95% *CI*: 0.09, 0.45). When comparing these results to the original serial mediation model described in the main text, we believe that attitude correctness doesn’t play as large of a role in the hypothesized paths from value basis to resistance success as attitude clarity does. Similarly, when the same model is conducted (i.e., replacing attitude clarity with attitude correctness) with intentions as the outcome, results reveal that subjective elaboration mediates the path between the value basis manipulation and intentions (*b* = 0.12, *SE* = 0.07, 95% *CI*: 0.01, 0.28), but all other confidence intervals for other indirect effects include 0. To summarize, across the two outcomes of resistance success and intentions, attitude clarity plays a greater role in value basis effects on the outcomes through perceived elaboration.

7 Because we included a measure of global certainty, we were able to test, consistent with previous research whether attitude clarity and correctness have independent effects on global certainty. Using methods similar to Petrocelli et al. (2007) and Cheatham and Tormala (2015), we conducted a series of mediation analyses (PROCESS Model 4; Hayes, 2013) to examine this possibility. We first conducted an analysis with value basis was the distal variable, attitude clarity as the mediator, and global certainty as the dependent measure, with attitude correctness as a covariate. Results revealed that the indirect effect of attitude clarity on global certainty did not include 0 [*b* = 0.19, *SE* = 0.08, 95% *CI*: 0.06 to 0.35], and the direct effect of value basis on global certainty was no longer significant [*b* = −0.04, *SE* = 0.09, *t*(146) = −0.5, *p* = 0.62, 95% *CI*: −0.22 to 0.13]. An alternative mediation model with attitude correctness as the potential mediator and attitude clarity as the covariate revealed that the indirect effect of attitude correctness on global certainty did include 0 [*b* = −0.01, *SE* = 0.02, 95% *CI*: −0.06 to 0.02]. Thus, although tentative, perceptions that one’s attitude is strongly based in values may be an additional antecedent of attitude clarity, which further distinguishes it from attitude correctness.
